# Theoretical predictions for hot-carrier generation from surface plasmon decay

**DOI:** 10.1038/ncomms6788

**Published:** 2014-12-16

**Authors:** Ravishankar Sundararaman, Prineha Narang, Adam S. Jermyn, William A. Goddard III, Harry A. Atwater

**Affiliations:** 1 Joint Center for Artificial Photosynthesis, California Institute of Technology, 1200 East California Boulevard, Pasadena, California 91125, USA; 2 Thomas J. Watson Laboratories of Applied Physics, California Institute of Technology, 1200 East California Boulevard, Pasadena, California 91125, USA; 3 Division of Physics, Mathematics and Astronomy, California Institute of Technology, 1200 East California Boulevard, Pasadena, California 91125, USA; 4 Materials and Process Simulation Center, California Institute of Technology, 1200 East California Boulevard, Pasadena, California 91125, USA

## Abstract

Decay of surface plasmons to hot carriers finds a wide variety of applications in energy conversion, photocatalysis and photodetection. However, a detailed theoretical description of plasmonic hot-carrier generation in real materials has remained incomplete. Here we report predictions for the prompt distributions of excited ‘hot’ electrons and holes generated by plasmon decay, before inelastic relaxation, using a quantized plasmon model with detailed electronic structure. We find that carrier energy distributions are sensitive to the electronic band structure of the metal: gold and copper produce holes hotter than electrons by 1–2 eV, while silver and aluminium distribute energies more equitably between electrons and holes. Momentum-direction distributions for hot carriers are anisotropic, dominated by the plasmon polarization for aluminium and by the crystal orientation for noble metals. We show that in thin metallic films intraband transitions can alter the carrier distributions, producing hotter electrons in gold, but interband transitions remain dominant.

## Abstract (web summary)

A full theoretical understanding of plasmon decay into hot carriers will help in applications such as solar cells or photocatalysis. Here, the authors present a quantized plasmon model to calculate the hot-carrier distribution from plasmon decay and show its sensitivity to the band structure of the host metal.

## Graphical abstract

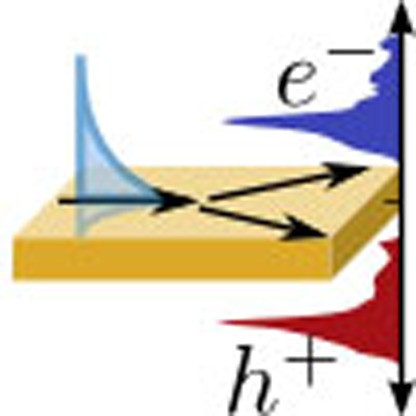

Plasmons are collective oscillations of electrons that couple to electromagnetic fields. They exhibit wave-like as well as particle-like behaviour^1^, support intense electromagnetic field concentrations^2^ and provide a pathway to couple optical energy from free space in nanoscale systems^3^. Surface plasmons, electromagnetic modes confined to the surface of a conductor–dielectric interface, have sparked recent interest because of their quantum nature^4^,^5^ and their broad range of applications, including solar energy harvesting^6^, nonlinear optics, tunable photodetectors^7^ and spectroscopy. Decay of plasmons to hot carriers has recently attracted considerable interest^8^ due to applications in energy conversion, photocatalysis and photodetection^9^.

Surface plasmons can decay either radiatively^10^ via emission of a photon or non-radiatively through the generation of excited carriers, typically referred to as hot carriers. These photo-excited hot carriers in metals could be used to directly drive energetically demanding chemical reactions^11^, or they could be transferred to a semiconductor for use in photovoltaics^12^,^13^ and photoelectrochemical systems^6^,^14^. At a metal–semiconductor interface, plasmonic hot-carrier collection over a tunable Schottky barrier allows the collection of photons with energies lower than the interband threshold of the semiconductor, thereby enabling additional energy harvesting. These excited carriers, both electrons and holes, can be injected into other materials, for example, graphene^15^ and MoS_2_ (ref. 16), thereby enabling plasmonic hot-carrier-induced doping and phase transitions.

Despite the significant experimental work in this direction, a complete theoretical understanding of plasmon-driven hot-carrier generation with electronic structure details has been evasive. Understanding the initial energy distribution of carriers generated by plasmon decay, before inelastic relaxation, is the first key step towards exploiting these phenomena. Theoretical studies of plasmonic systems have traditionally focused on their optical response, including quantum jellium models of nanostructured systems such as nanoparticle dimers^17^,^18^,^19^,^20^, and detailed time-dependent density functional calculations of short-wavelength surface plasmons on noble metal surfaces^21^. Recently, the initial hot-carrier distribution generated by plasmon decay has been estimated within a simple electron gas model for various geometries^22^ and within a jellium model for silver nanoparticles and nanoshells^23^. These models provide insight into the mechanisms of plasmonic hot-carrier generation, but do not capture the material dependence of this process and miss interband transitions in noble metals since they preclude transitions involving *d* bands.

In this article, we combine quantized plasmon modes from experimental dielectric functions with electronic states from first principles density functional theory (DFT) to calculate the initial distribution of hot carriers in real materials. We first examine the direct electron excitations generated by the decay of surface plasmon polaritons on planar metal–dielectric interfaces, as shown schematically in Fig. 1a. (Note that the surface plasmon and the initial photon have the same energy, and a coupling geometry such as a grating provides the change in momentum^24^.) This allows us to explore the effects of the electronic structure of the metal on the generated carrier distributions, independent from other effects such as geometry. In addition, we focus on interband transitions that dominate at higher plasmon energies since these are expected to be more sensitive to the electronic structure than intraband transitions; the latter dominate at lower plasmon energies and have been described within simplified jellium models^23^. Finally, we analyse the effects of geometry on the generated hot-carrier distribution in real materials by studying the decay of plasmon modes in thin metallic films of varying thickness.

## Results

### Interband transition rate formalism

We describe the surface plasmon using an explicit quantization of the surface modes^25^,^26^,^27^ derived from an experimental dielectric function^28^. The vector potential operator for plasmons on the surface of a semi-infinite slab (with normal along the *z* direction) is 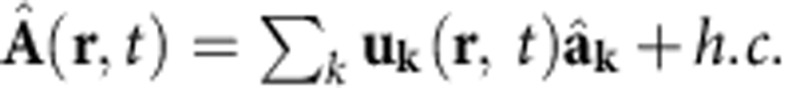, in terms of creation and annihilation operators, 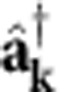 and 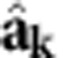, and the normalized mode functions of wave vector **k** and angular frequency *ω*,


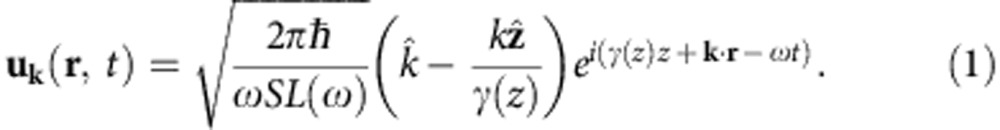


The modes satisfy the dispersion relation 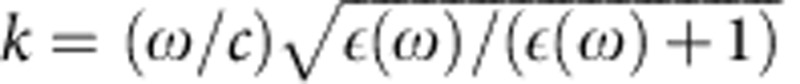, where *ε*(*ω*) is the experimental dielectric function of the metal. The *z* wavenumber satisfies 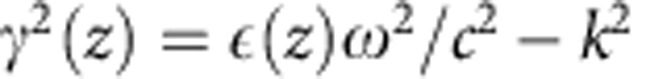, where *ε*(*z*)=*ε*(*ω*) for *z*<0 and 1 and for *z*>0, and with the sign of Im*γ* (*z*) set so that the modes decay away from the surface. Above, *S* is a test area with periodic boundary conditions for discretizing the modes and *L*(*ω*) is a normalization length chosen so that each mode has energy *ħω*. (See ref. 26 for details.) Although this neglects the possibility of nonlocal effects in the dielectric matrix, such an approximation is valid at the wavelengths of interest.

Next, given an approximation of the quasiparticle orbitals 
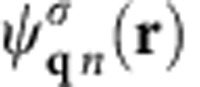
 and energies *ε*_**q***n*_ of the metal, the electron field operator is 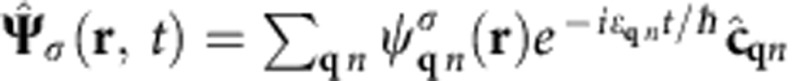. Here, 
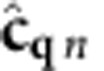
 and 
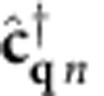
 are Fermionic creation and annihilation operators for electrons with wave vector 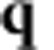 and band index *n*. We have included the spinor index *σ* in the orbitals to fully treat relativistic effects such as spin-orbit coupling, when necessary.

Finally, we approximate the plasmon–quasiparticle interaction Hamiltonian using the lowest order (unrenormalized) vertex, 
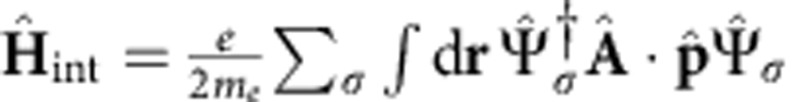
, where 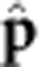 is the electronic momentum operator. Fermi’s golden rule for the decay of a single plasmon with wave vector **k** and angular frequency *ω* to electron–hole pairs via interband transitions then reduces to


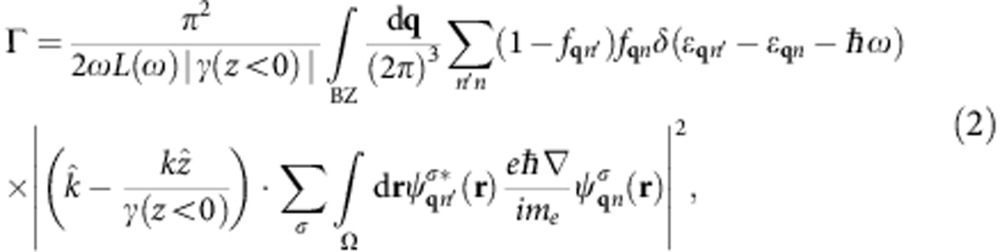


where *f*_**q***n*_ are the occupation factors of the quasiparticles in the Fermi sea. (See the Methods section for a brief derivation.) The key approximation above is that the plasmon mode function varies slowly on the atomic scale (interband approximation).

### Electronic structure method selection

To calculate the carrier distributions from plasmon decays in real materials using equation (2), we need a sufficiently accurate prescription for the quasiparticle orbitals and energies. Figure 2 compares the accuracy of different electronic structure methods for the noble metals copper, silver and gold. The different methods produce identical results for aluminium since it is a nearly free electron metal; we omit that comparison for brevity and select the PBEsol generalized-gradient approximation^29^ within DFT.

In contrast, for the noble metals, generalized-gradient approximations such as PBEsol predict the *d* band positions to be closer to the Fermi level than that of experiment because of the self-interaction error for localized electrons. The GLLB-sc orbital-dependent functional^30^ partially remedies this situation, as shown by a recent density functional study of plasmon dispersions on noble metal surfaces^21^. However, Fig. 2 shows that the band structure predicted by this functional still exhibits significant deviations from angle-resolved photoemission measurements^31^,^32^,^33^. These deviations are largest (~0.5 eV) near the *L* point in the Brillouin zone, and this region of the Brillouin zone is particularly active for interband transitions in these metals. Many-body perturbation theory methods, such as the quasiparticle self-consistent *GW* approximation, significantly improve the agreement with experiment as shown for gold in Fig. 2c (*GW* results from Rangel *et al*.^34^), however, the error near the *L* point remains large (~0.5 eV).

We find that the DFT+*U* method^35^, which improves the description of localized electrons with a local semi-empirical correction on each atom, yields the best agreement (~0.1 eV) to the quasiparticle bandstructure of all three noble metals (indicated by PBEsol+*U* in Fig. 2, since we combine the *U* correction with the PBEsol density functional). We pick the value of *U* to reproduce the experimental energies at the Γ point, which results in *U*=1.63, 2.45 and 2.04 eV for copper, silver and gold, respectively.

The calculations presented here account for relativistic effects including spin-orbit coupling fully self-consistently. The spin-orbit splitting at the *X* point is particularly relevant, since it determines the inter-band threshold energy. The magnitude of the effect is ~0.5 eV in gold, ~0.2 eV in silver and ~0.1 eV in copper. Therefore, the inclusion of spin-orbit splitting is critical in calculations of optical transitions in gold, still quite significant for silver and negligible at the achievable accuracy for copper (and aluminium).

### Hot-carrier distributions from surface plasmon decay

Substituting the electronic states and energies from the PBEsol+*U* density functional method in equation (2) allows us to predict accurate carrier distributions. We histogram the contributions to the decay rate Γ by the electron (final state) and hole (initial state) energies to generate the distributions shown in Fig. 3 and by the carrier energies as well as momentum directions to generate Fig. 4. Energy conservation determines which regions of the Brillouin zone contribute to the plasmon decay, and the top panels of Fig. 3 annotate the allowed transitions for each metal at selected energies.

For aluminium, the band crossing close to the Fermi level near the W point allows interband transitions that originate from valence band states with energies ranging continuously from the Fermi level to *ħω* below it. Consequently, surface plasmon decay results in both hot electrons and holes with continuous energy distributions that extend from zero energy to the plasmon energy, as seen in the bottom panel of Fig. 3a. Additional transitions near the K point contribute predominantly to hot holes with energies >2 eV and lead to the moderate asymmetry between the electron and hole energy distributions.

For silver, the lowest energy interband transitions, originating from the *d* bands at the X and L points as well as from the Fermi level at the L point, all appear at ~3.6 eV. Consequently, the decay of a plasmon of that energy produces bimodal energy distributions for both the electrons and holes as shown in Fig. 3b.

In copper and gold, the allowed interband transitions near the resonant surface plasmon polariton energies occur near the X and L points for both metals, and additionally near the K point for copper, as shown in Fig. 3c,d. Notice that all these transitions originate in the *d* bands that are approximately *E*_t_=2 eV below the Fermi level. Consequently, for these metals, the generated holes are on average more energetic than the electrons by *E*_t_.

**Figure 1:**
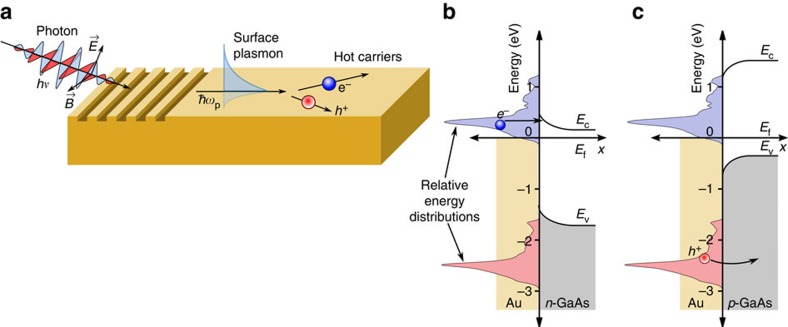
Schematic of plasmonic hot-carrier generation and injection. (**a**) Schematic for optical excitation of surface plasmons followed by decay to hot carriers, (**b**) tunnelling of plasmonic hot electrons from gold through a Schottky barrier into *n*-type gallium arsenide using the predicted carrier distribution from Fig. 3d and typical experimental band offsets^40^ and (**c**) barrier-less injection of plasmonic hot holes from gold into *p*-type gallium arsenide. (*E*_f_ is the Fermi energy, *E*_c_ the conduction band minimum and *E*_v_ the valence band maximum energy.)

**Figure 2:**
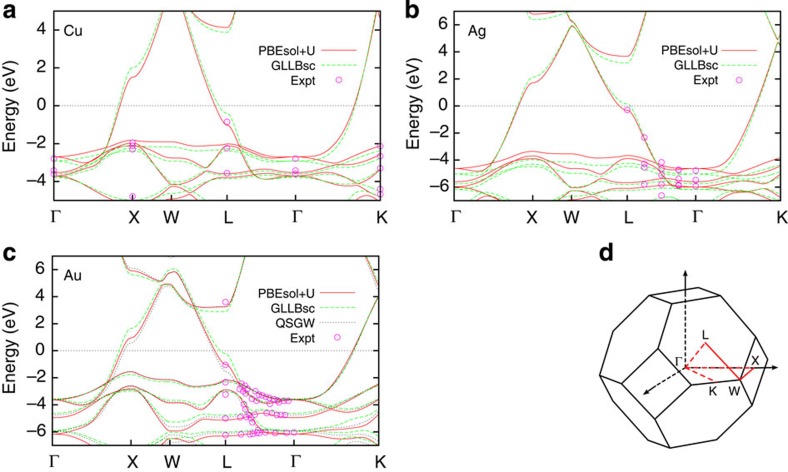
Comparison of theoretical and experimental band structures. Theoretical band structures for (**a**) copper, (**b**) silver and (**c**) gold as predicted by different density functional approximations, compared with angle-resolved ultraviolet photoemission measurements^31^,^32^,^33^ and quasiparticle self-consistent *GW* calculations^34^. (**d**) The high-symmetry paths in the Brillouin zone along which the band structures are plotted. All calculations account for relativistic effects including spin-orbit coupling. The PBEsol+*U* approximation, with *U* fit to the experimental Γ point energies, provides the best overall agreement with the experimental data for all three noble metals and particularly improves on the accuracy of the other methods near the L point.

**Figure 3:**
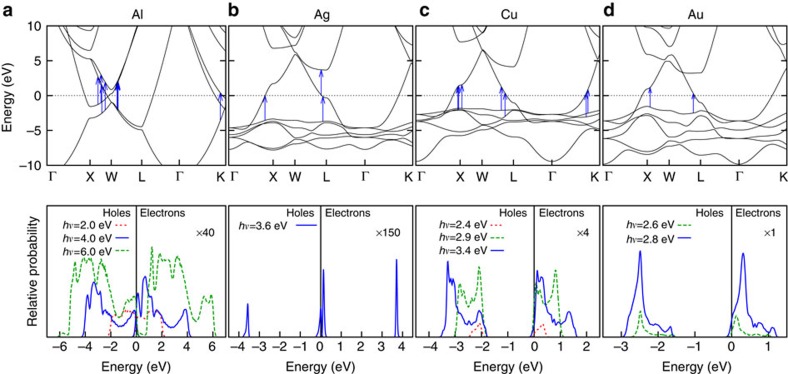
Allowed transitions and hot-carrier energy distribution. PBEsol+*U* band structure and predicted plasmonic hot-carrier energy distributions for (**a**) aluminium, (**b**) silver, (**c**) copper and (**d**) gold. The bottom panels show the energy distribution of hot electrons (positive energies relative to Fermi level at 0) and hot holes (negative energies) for various photon and plasmon energies, *hν*. The top panels show the band structure and arrows mark the allowed transitions for the plasmon energy plotted with a solid line in the corresponding bottom panel. Contrast the almost uniform energy distribution of electron and hole energies in aluminium with the hole-dominant energy distribution in copper and gold and the bimodal hot-hole and hot-electron distributions in silver due to the position of the *d* bands.

The asymmetry between energy distributions for plasmonic hot electrons and holes in copper and gold has important consequences for collection efficiencies across a metal–semiconductor interface. Consider, for example, the gold to *n*-type gallium arsenide Schottky junction in Fig. 1b, which includes the predicted hot-carrier distributions from Fig. 3d. Most of the hot electrons are not sufficiently energetic to overcome the Schottky barrier, and would either have to tunnel through the barrier or would require an additional thermal boost to overcome it. In contrast, in the corresponding junction to *p*-type gallium arsenide shown in Fig. 1c, all the holes are sufficiently energetic to cross into the semiconductor. This barrier-less collection of holes would exhibit significantly higher efficiency and would require lower biases than electron collection. Combining the results of Fig. 3 with this picture, gold and copper are ideally suited for hot hole injection, whereas silver and aluminium are capable of both.

**Figure 4:**
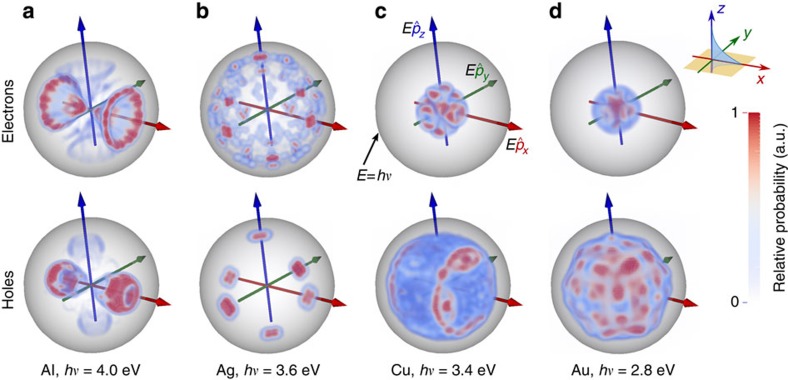
Hot-carrier energy and momentum-direction distribution. Plasmonic hot-carrier energy and momentum-direction distribution in (**a**) aluminium (**b**) silver (**c**) copper and (**d**) gold. The radial coordinate in each panel is the carrier energy relative to the Fermi level, with the spherical shell indicating the plasmon (photon) energy, *hν*, while the angular coordinates correspond to the carrier momentum direction. The asymmetry in electron and hole energies in the noble metals from Fig. 3 is manifested in the radial extent of the corresponding probability clouds here. None of the metals exhibit the isotropic orientation distribution assumed in the Fowler theory.

The nature of transitions accessible by the plasmon also affects the angular distribution of the excited carriers. For the four metals considered above, Fig. 4 illustrates the momentum direction and energy distribution for hot carriers generated by the decay of a surface plasmon polariton propagating on a [001] surface along the *x* direction. The radial direction indicates carrier energy, whereas the orientation indicates the carrier momentum direction. The maximum energy, illustrated by the grey sphere, corresponds to the photon energy, and the colour scheme indicates the relative probability density of carriers at that energy and momentum direction.

The electric field due to such a plasmon is predominantly along the *x* direction, and for a free-electron metal we expect a dipole-antenna-like momentum distribution that peaks along the field directions. Only the electron and hole distributions in aluminium exhibit such an orientation dependence; the remaining metals deviate significantly from that idealized prediction. For the noble metals, the allowed transitions are on a surface in *k* space containing the X and L points, which contributes carriers in all directions, but with a strong anisotropy dominated by the crystal directions rather than the plasmon field.

The collection efficiency of hot carriers in plasmonic structures depends on both the initial distribution and transport of the carriers. In faceted structures smaller than the carrier mean free path, ballistic transport, which preserves the momentum direction, is significant compared with diffusive transport. The crystal orientation-dependent anisotropy in the initial momentum distribution would therefore become particularly important for such structures.

### Geometry effects in decay of thin-film plasmons

Above we considered the decay of surface plasmon polaritons on semi-infinite metal slabs to minimize geometry effects and focus on the effects of electronic structure. In nano-confined geometries, crystal momentum or 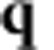 is no longer a good quantum number due to the uncertainty principle and therefore the transitions excited by plasmon decay no longer need to be vertical unlike the situation in Fig. 3. This opens up the possibility of geometry-assisted intraband transitions without involving phonons. This mode of localized plasmon decay has been studied within the context of jellium models with simplified electronic structure^22^,^23^; here we analyse this decay mode, including the full electronic structure of the plasmonic metal.

Direct electronic structure calculations for nanoparticles require significant computational effort; however, and we therefore make two simplifications to enable practical calculations for systems of experimentally relevant sizes. First, we consider thin films that are of finite thickness along one dimension; the electronic structure calculation can then exploit Bloch’s theorem in the remaining two periodic directions. The resulting predictions would then be a lower bound on the corresponding geometry effect in nanoparticles, which are confined in all three dimensions. Second, we adopt an *ab initio* tight-binding approximation for the electronic structure of the thin film using the density functional Hamiltonian expressed in the basis of maximally localized Wannier functions^36^,^37^. This approach reproduces the full density functional band structure of the bulk material exactly by construction, but approximates geometry effects since changes in orbital shapes within a unit cell are neglected.

**Figure 5:**
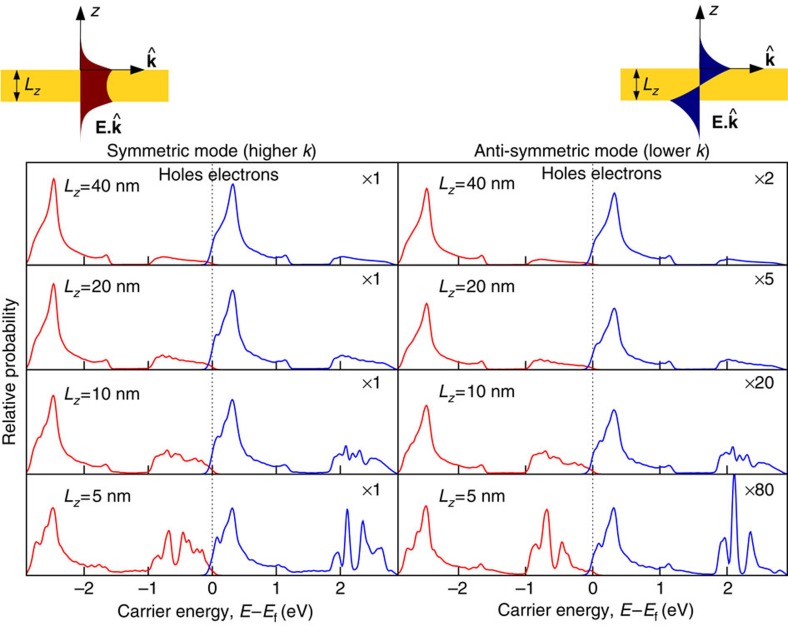
Energy distribution of hot carriers from thin-film plasmon decay. Energy distributions of hot carriers (electrons in blue, holes in red) generated by the decay of symmetric and antisymmetric plasmon modes of energy 2.8 eV on gold thin films of various thicknesses. As the film thickness decreases, the relative probability for generating hot electrons via geometry-assisted intraband transitions (compared with hot holes generated via interband transitions) increases.

A metal film of finite thickness supports symmetric and antisymmetric plasmon modes (with respect to 
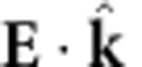
, the electric field along the propagation direction) as shown schematically in the top insets of Fig. 5. We analytically construct the vector potential for one quantum of each of these modes^38^, analogous to equation (1), and use Fermi’s golden rule to calculate the rate of their direct decay to electron–hole pairs with the matrix elements and electronic energies obtained from the Wannier representation. See the Methods section for details.

Figure 5 shows the resulting electron and hole energy distributions generated from the decay of plasmons in gold films. The results for both the symmetric and antisymmetric plasmon modes on a film of thickness 40 nm resemble that for the semi-infinite slab in Fig. 3d: most of the energy is deposited in the hot holes. However, with decreasing film thickness, the finite-thickness geometrical effects become stronger and the probability of generating hot electrons via intraband transitions increases. The effect is particularly pronounced for the antisymmetric mode because this lower wave vector mode becomes more light-like with a smaller fraction of the field in the interior of the metal, which lowers the contribution of the interband transitions (note the scale factors in the right hand panels of Fig. 5).

This analysis allows one to weigh the relative importance of the interband transitions and geometry-assisted intraband transitions. Confining geometries smaller than 10 nm enable finite probabilities of intraband transitions and allow the generation of hotter electrons than allowed in the hole-dominant bulk copper or gold. However, interband transitions are still responsible for a significant fraction of the generated carriers, and hence an appropriate choice of material (as discussed in the previous subsection) is important to maximize the efficiency of hot electron or hole generation.

## Discussion

We have reported first principles calculations that describe plasmon-mediated hot-carrier generation in aluminium, gold, silver and copper. These calculations illustrate that the generated carrier profile is extremely sensitive to the details of the electronic band structure, especially to the position of the *d* bands in silver, copper and gold relative to unoccupied states above the Fermi level. Copper and gold generate hot holes that are much more energetic than the electrons, silver produces narrow energy distributions of hot holes as well as hot electrons, while aluminium generates continuous energy distributions of holes and electrons. These findings inform material selection for efficiently collecting carriers of a specific type and energy at metal–semiconductor interfaces or in surface-adsorbed molecular species.

Geometry of the plasmonic structure also plays an important role in determining the efficiency of carrier generation and collection. Nano-confinement effects allow the generation of hotter electrons in copper and gold via geometry-induced intraband transitions. However, interband transitions, which depend strongly on the electronic band structure, still dominate the initial energy distribution. The initial momentum distribution of the carriers depends on both the crystallographic orientation of the metal and plasmon polarization. The net efficiency of carrier collection in a specific geometry depends on this initial distribution as well as the subsequent transport of the carriers to the surface. Therefore, assessing and optimizing carrier collection efficiency of plasmonic nano-structures additionally require models for the transport of hot carriers, a subject for future study.

## Methods

### Computational details

We perform density functional calculations for face-centred cubic aluminium, silver, copper and gold in the plane-wave electronic density functional software, JDFTx (ref. 39), using full-relativistic norm-conserving pseudopotentials at a kinetic energy cut-off of 30 hartrees (816 eV) and at the experimental lattice geometry. We use the PBEsol^29^ exchange-correlation approximation along with a rotationally invariant DFT+*U* correction^35^ for the *d* electrons, with *U*=1.63 eV for copper, 2.45 eV for silver and 2.04 eV for gold fit to reproduce experimental photoemission data (no *U* correction for aluminium).

We use Kohn–Sham eigenvalues, Fermi occupations and momentum matrix elements on a dense 128^3^ sampling of the Brillouin zone to calculate the inter-band transition rate of the plasmon mode given by equation (1) using equation (2). We use experimental dielectric functions from ref. 28, parametrized as a sum of Lorentz–Drude responses, to determine the plasmon mode. We replace the energy-conserving *δ* function in equation (2) with a normalized Gaussian of width ~*k*_B_*T*=0.026 eV to accommodate the discrete *k* point sum that replaces the Brillouin zone integral. We histogram the contributions to the total transition rate Γ in terms of the electron and hole energies and momenta to generate the distributions plotted in Figs 3 and 4.

For the thin-film calculations, we generate the Wannier functions, Hamiltonian and matrix elements using a coarser 10^3^ sampling of the Brillouin zone, which results in an *ab initio* tight-binding-like Hamiltonian with a range of ~5 unit cells in each direction. We then calculate the electronic states of the thin film in the Wannier basis and calculate the transition rate of the plasmon modes given by equations (8)–(13), 33, 36, 37, 39, 40 using equation (14), histogrammed by electron and hole energies to generate the distributions shown in equation (4).

### Surface plasmon polariton decay rate

Here we sketch the derivation of equation (2), the decay rate of a single surface plasmon to electron–hole pairs via interband transitions. The initial state for this decay is the Fermi sea of quasiparticles and a single surface plasmon with wave vector **k**, which we can denote by 
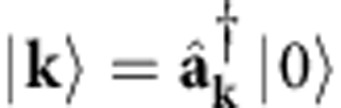
, if we define |0› to consist of the vacuum of surface plasmons and the Fermi sea. The possible final states each consist of a single electron–hole pair on the Fermi sea and no plasmon, 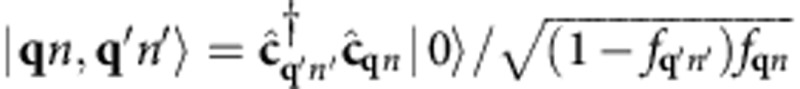, and are therefore labelled by the electron and hole wave vectors **q**, **q′** and band indices *n*, *n*′. (The occupation factors normalize the final states, since 
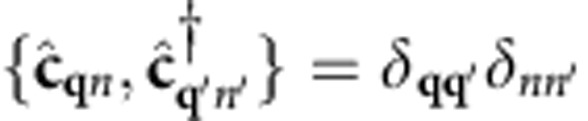
 and 
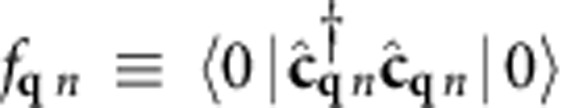
.)

Fermi’s golden rule for this decay process is therefore


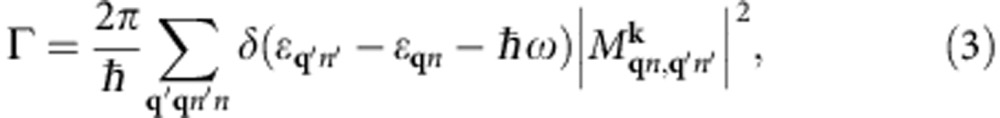


with the transition matrix element


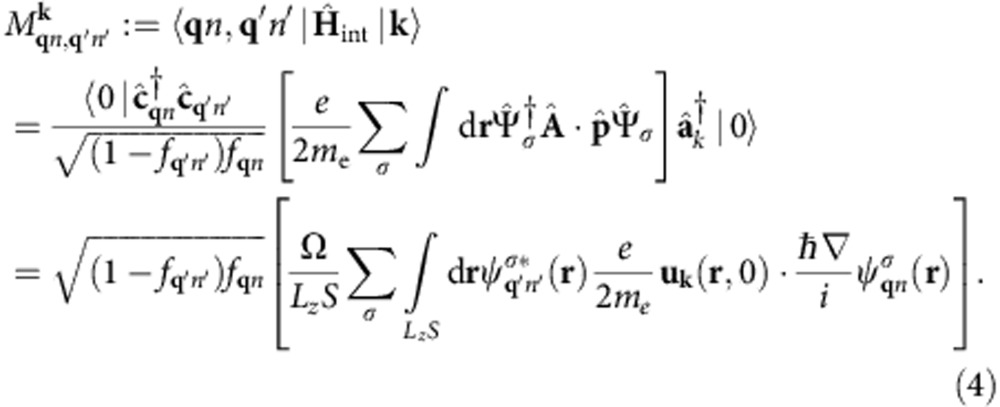


The expectation value of the plasmon and quasiparticle creation and annihilation operators in the second line above against the vacuum and Fermi sea reduces to (1−*f*_**q**′*n*′_) *f*_**q***n*_. In the final line above, *S* is the surface area for plasmon quantization^26^, and we quantize the electrons on a box of area *S* on the surface that extends a depth *L*_*z*_ into the surface, with *L*_*z*_>>1/|*γ*(*z*<0)|, the decay length of the plasmon mode in the metal. The factor Ω/(*L*_*z*_*S*) above accounts for the fact that the orbitals are normalized on the unit cell of volume Ω instead of on the quantization volume.

Substituting the plasmon mode function given by equation (1), noting that it varies slowly on the length scale of the orbitals (both **k** and |*γ*(*z*<0)| are small in atomic units) and splitting 
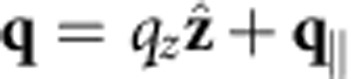
, the normal- and surface-plane components, the matrix element reduces to


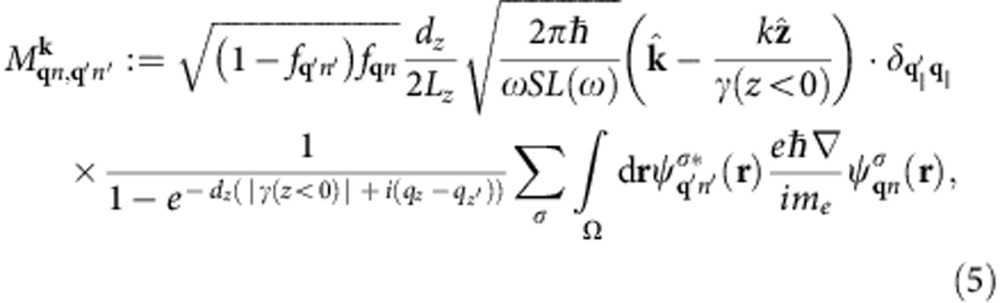


where *d*_*z*_ is the separation between lattice planes. The term with the exponential in the denominator arises from the sum of a geometric series over lattice planes and is a sharply peaked function of *q*_*z*_−*q*_*z*′_ with width ~|*γ*(*z*<0)|. We can therefore approximate it by 
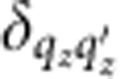
 in the total transition rate with weight equal to


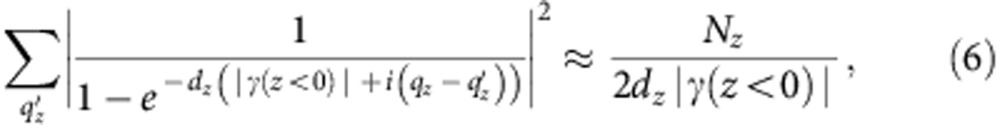


where *N*_*z*_=*L*_*z*_/*d*_*z*_ is the number of lattice planes in the quantization volume. Reducing the double sum over **q** using 
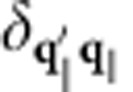
 and 
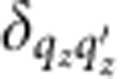
, the total transition rate simplifies to


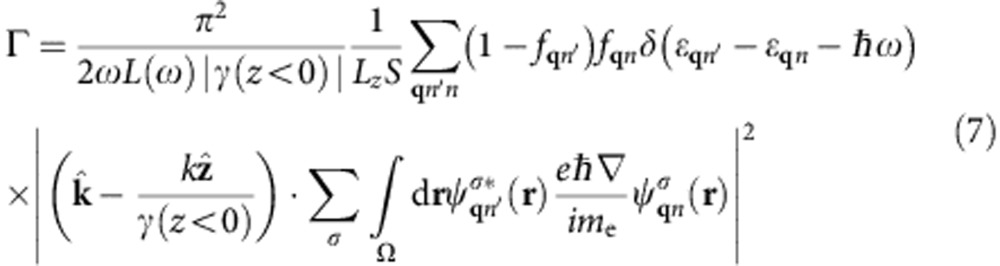


Finally, replacing the discrete average over wave vectors 
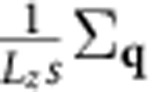
 by its continuum limit 
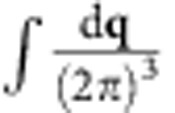
 gets rid of all dependence on the fictitious plasmon quantization area and electron quantization volume, and results in equation (2) in the main text.

### Thin-film plasmon decay rate

Here we briefly sketch the construction of the plasmon mode functions, the Wannier basis approximation to the electronic states and the Fermi golden rule calculation for the surface plasmon decay in thin metallic films (as shown in Fig. 5).

Given a thin metal film of thickness *L*_*z*_=2*H* centred at *z*=0 described by a local dielectric function *ε* (*ω*), we can solve Maxwell’s equations analytically to obtain the symmetric mode


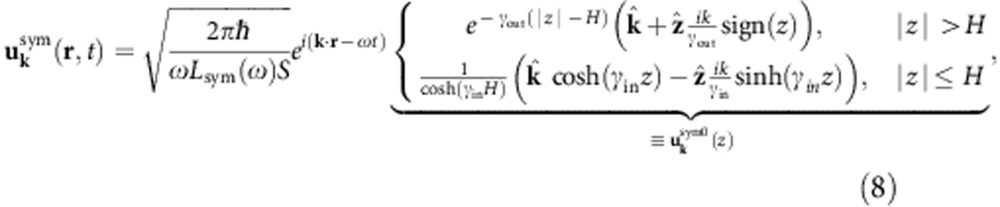


and the antisymmetric mode


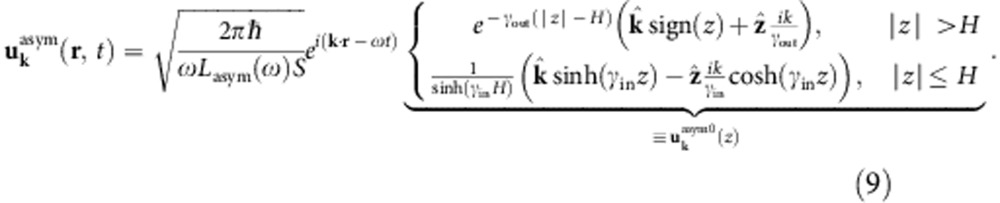


Here, 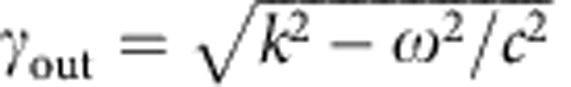 and 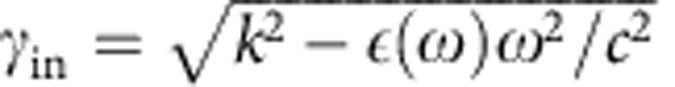, where *k* satisfies the dispersion relation


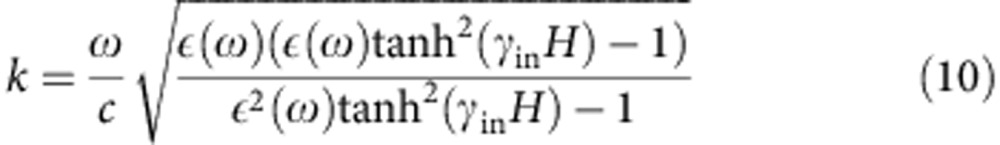


for the symmetric mode and


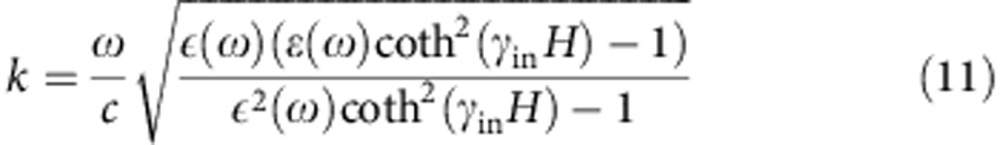


for the antisymmetric mode (obtained by imposing continuity of 
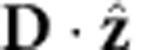
 across the interface).

Above, the prefactors in equations (8) and (9) normalize the energy of the plasmon modes on an area *S* to that of a single quantum, *ħω*. (See ref. 38 for more details about quantization of surface plasmons on thin films.) Integrating the field intensities and enforcing this normalization then results in


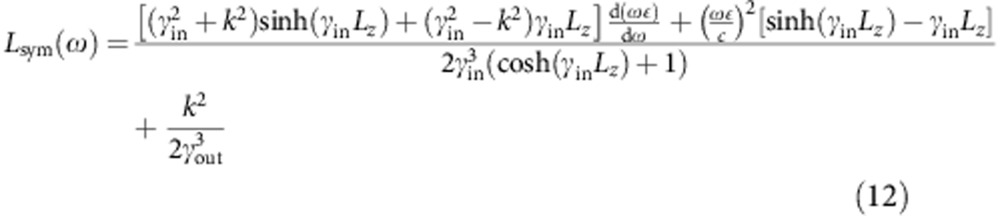


for the symmetric mode and


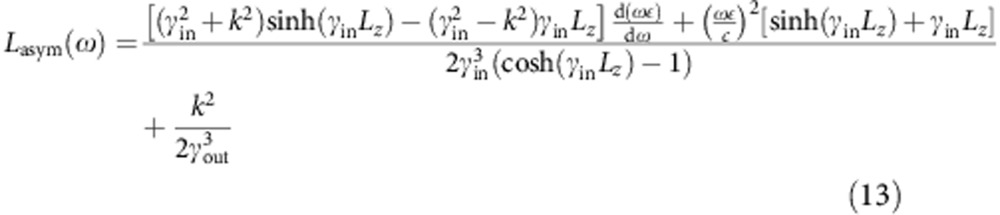


for the antisymmetric mode.

As for the electronic states, we start by computing the maximally localized Wannier functions^36^,^37^ 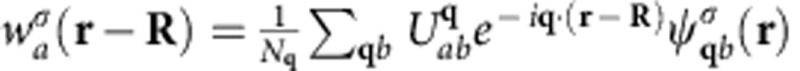 for the bulk metal, which involves finding the unitary rotations 
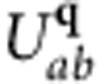
 that minimize the spatial variance of 
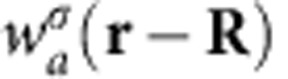
. Here, 
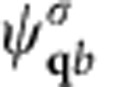
 are the eigen orbitals of the bulk metal and let *ε*_**q***b*_ be the corresponding eigen energies. Now, we can use the unitary transformations to construct the Hamiltonian in the basis of Wannier functions, 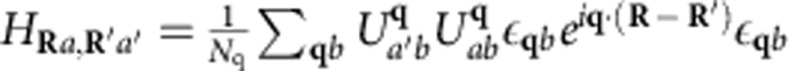. The fact that the Wannier functions are localized then implies that the elements of *H*_**R***a*,**R′***a*′_ decrease rapidly with increasing |**R***−***R′**| and can be truncated after a finite number of sites. This is therefore a multi-orbital tight-binding-like model (with many neighbours) that exactly reproduces the original eigenfunctions and eigenvalues since it is obtained from a unitary transformation of the original Hamiltonian. See ref. 37 for a detailed exposition.

Next we approximate the Hamiltonian for the thin film by starting with the above Wannier basis Hamiltonian for the bulk system and setting any matrix elements that involve a site outside the film to zero. Diagonalizing this discrete Hamiltonian then gives us the eigenvalues *ε*_**q***n*_ and eigenfunctions 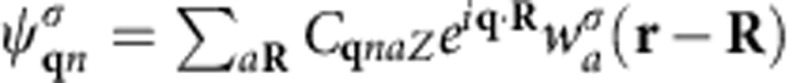 as a linear combination of the localized Wannier functions on various atom sites **R**. Here we have used Bloch’s theorem in the two periodic directions to obtain the diagonalizing factor *e*^*i***q·R**^, where **q** is a wave vector in the two-dimensional (2D) Brillouin zone. The coefficients *C*_**q***naZ*_ (where 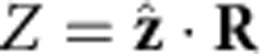) are obtained from numerical diagonalization of the discrete Hamiltonian matrix constructed above.

Finally, we apply Fermi’s golden rule to calculate the transition rates using the vector potentials for the thin-film plasmon modes and the eigenfunctions in the Wannier basis. Assuming that 
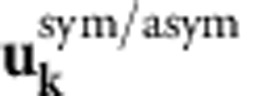
 vary slowly on the atomic scale in the two periodic directions, we can show analogously to the previous section that the transition rates for the symmetric/antisymmetric modes are


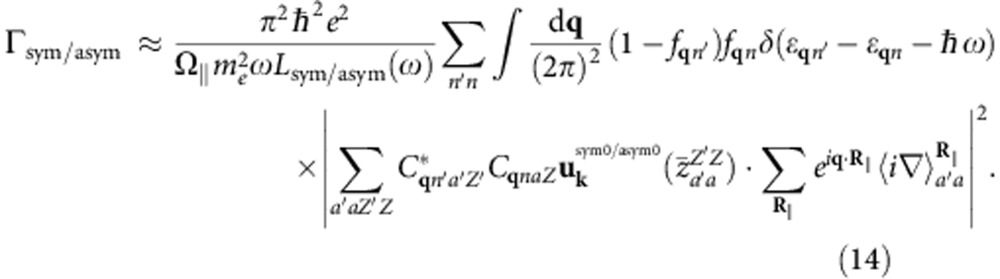


Here, Ω_||_ is the area of the 2D surface unit cell, 
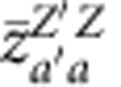
 is the *z* centre of the product density of the Wannier basis functions indexed by *aZ* and *a*′*Z*′ and 
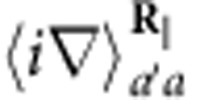
 is the momentum matrix element between Wannier functions *a* and *a*′ at two sites separated by **R**_||_, a lattice vector parallel to the surface. As before, we generate the carrier energy distributions by histogramming contributions to the integral in the above expression in terms of the initial and final electronic state energies.

## Author contributions

All authors contributed to all aspects of this work.

## Additional information

**How to cite this article:** Sundararaman, R. *et al*. Theoretical predictions for hot-carrier generation from surface plasmon decay. *Nat. Commun.* 5:5788 doi: 10.1038/ncomms6788 (2014).
